# The Alcohol Environment Protocol: A new tool for alcohol policy

**DOI:** 10.1111/dar.12654

**Published:** 2018-01-04

**Authors:** Sally Casswell, Neo Morojele, Petal Petersen Williams, Surasak Chaiyasong, Ross Gordon, Gaile Gray‐Phillip, Pham Viet Cuong, Anne‐Marie MacKintosh, Sharon Halliday, Renee Railton, Steve Randerson, Charles D. H. Parry

**Affiliations:** ^1^ SHORE & Whariki Research Centre Massey University Auckland New Zealand; ^2^ UK Centre for Tobacco & Alcohol Studies Nottingham UK; ^3^ Alcohol, Tobacco and Other Drug Research Unit Medical Research Council Pretoria South Africa; ^4^ Department of Psychiatry and Mental Health University of Cape Town Cape Town South Africa; ^5^ Health Promotion Policy Research Center International Health Policy Program Nonthaburi Thailand; ^6^ Social Pharmacy Research Unit, Faculty of Pharmacy Mahasarakham University Maha Sarakham Thailand; ^7^ Department of Marketing and Management, Faculty of Business and Economics Macquarie University Sydney Australia; ^8^ St. Kitts‐Nevis National Council on Drug Abuse Prevention Secretariat Basseterre St. Kitts and Nevis; ^9^ Hanoi University of Public Health Hanoi Vietnam; ^10^ Institute for Social Marketing University of Stirling Stirling UK; ^11^ RAPHA Healthcare Services North Carolina Durham USA; ^12^ Department of Psychiatry Stellenbosch University Cape Town South Africa

**Keywords:** alcohol policy, international comparison, policy analysis, protocol

## Abstract

**Introduction and Aim:**

To report data on the implementation of alcohol policies regarding availability and marketing, and drink driving, along with ratings of enforcement from two small high‐income to three high‐middle income countries, and one low‐middle income country.

**Method:**

This study uses the Alcohol Environment Protocol, an International Alcohol Control study research tool, which documents the alcohol policy environment by standardised collection of data from administrative sources, observational studies and interviews with key informants to allow for cross‐country comparison and change over time.

**Results:**

All countries showed adoption to varying extents of key effective policy approaches outlined in the *World Health Organization Global Strategy to Reduce the Harmful Use of Alcohol* (2010). High‐income countries were more likely to allocate resources to enforcement. However, where enforcement and implementation were high, policy on availability was fairly liberal. Key Informants judged alcohol to be very available in both high‐ and middle‐income countries, reflecting liberal policy in the former and less implementation and enforcement and informal (unlicensed) sale of alcohol in the latter. Marketing was largely unrestricted in all countries and while drink‐driving legislation was in place, it was less well enforced in middle‐income countries.

**Conclusion:**

In countries with fewer resources, alcohol policies are less effective because of lack of implementation and enforcement and, in the case of marketing, lack of regulation. This has implications for the increase in consumption taking place as a result of the expanding distribution and marketing of commercial alcohol and consequent increases in alcohol‐related harm.

## Introduction

### *Alcohol Environment Protocol*

The Alcohol Environment Protocol (AEP) is one of the two tools used in the International Alcohol Control (IAC) study 1, 2, 3. The AEP has been developed to allow countries to document and assess (in a comparable way) the environment, in which alcohol is sold and consumed, existing alcohol policies, levels of enforcement and document changes over time. The AEP includes sections covering availability (sale restrictions and social supply), drink‐driving enforcement and marketing/sponsorship. Less evidence‐based approaches, such as health warnings and drinking guidelines 4, are not included. The AEP is a tool for comparative policy analysis, which allows a focus on both policy inputs and policy impacts 5 and will allow policy surveillance and, in future work, examination of the relationship with consumption 6.

### *Participating countries*

This paper provides some illustrative data from six of the IAC countries using the AEP: Scotland, New Zealand, St. Kitts and Nevis, Thailand, South Africa and Vietnam (these are the countries for which data were available at the time of this analysis). The countries differ in terms of their current alcohol markets, including the proportions of the market comprising informal (untaxed alcohol) and recorded alcohol. They also differ in terms of their levels of economic development, histories of alcohol use and forms of government.

### *Methodology*

The AEP collects data on the legislative and regulatory aspects of alcohol policy and the degree of implementation and enforcement in each country. The AEP provides a structured framework to allow description (quantitative and qualitative) of the alcohol environment in different countries.

### *Data collection*

The time period covered by the AEP was 2013 and 2015. A mixed methods data collection approach included a review of policy and legislation documents, literature searches, observational surveys, administrative and commercial data sets and key informant interviews. Data were collected by local researchers in each country. The documentary data sources that were commonly used included: legislation and regulation; government documents and websites; parliamentary and executive publications; media reports and research reports. Observational surveys were used to document outlet density when licensing data were not available.

In relation to availability, data were collected separately for metropolitan areas (populations >30 000) and non‐metropolitan areas. This was relevant in four countries, but St. Kitts and Nevis had no towns with a population greater than 30 000 and non‐metropolitan areas were not covered in South Africa. Data were collected separately for off‐ and on‐premise, but countries differed in the extent to which a clear distinction between the two was part of policy or observable. For example, in Thailand the licensing system does not distinguish on‐premise from off‐premise.

Purposive sampling was used to ensure relevant key informants were selected including: policy makers, licensing board members, government and enforcement officers, police and other stakeholders responsible for formulating and advocating for alcohol policy. The numbers interviewed in each country varied from 12 in Scotland to 48 in South Africa. Each of the key informants gave a rating on a Likert scale from 1 (completely ignored/not enforced) to 10 (complete compliance/always enforced) of their perception of enforcement of various alcohol policies, and alcohol availability within their country. Open‐ended questions allowed for key informants to comment further on enforcement and policies.

### *Ethical approval*

Ethical approval to conduct the IAC study was obtained by each country.

### *Analysis*

Key informant data were collated into a spreadsheet and mean responses are presented to examine differences in policy and stakeholder perception across countries. Rates of outlet density were calculated per 1000 population.

### *Country contexts*

Scotland had a population of 5.4 million and is part of the United Kingdom, but has a devolved democratically elected government. The Scottish government has responsibility for health, justice and social affairs. Scotland has had its own alcohol‐licensing legislation dating back to the 18th century.

New Zealand had a population of 4.6 million and comprises two islands in the Southern Hemisphere. It was a British colony and is now an independent constitutional monarchy with a democratically elected parliament. The country is built on an 1840 treaty relationship between the government, representing the Crown and the indigenous people, Maori. Taxation on alcohol was very important in the early stages of the colony and alcohol legislation was drawn from the British tradition. The data were collected nationwide.

St. Kitts and Nevis, comprising two small islands in the Caribbean, had a population of only around 51 000 and the population mainly inhabits villages and towns. The population is predominantly Christian. Carnivals, sporting events and celebration play an important part in island life. A number of Acts regulate the licensing, sale and consumption of liquor and also the sale of alcohol to those under 18 years and their entry into establishments selling alcohol are prohibited. It is a parliamentary democracy with alcohol legislation drawing on the British colonial history. The study was carried out in both islands.

Thailand, a country of 65.7 million, is a constitutional monarchy that has experienced alternating periods of military government and of a democratic Westminster style parliament. The population retains strong affiliation to Buddhism, although the country is becoming more secular over time. Governments have had a strong intention to control alcohol‐related harm and delay the spread of alcohol use. The passing of the *Alcohol Control Act* in 2008 has been supplemented by a number of amendments 7. The government also established the Thai Health Promotion Foundation, funded by a levy on alcohol and tobacco sales that has addressed alcohol control. The study was carried out in five provinces from four regions including Bangkok covering a population of 11.5 million.

Vietnam is a socialist republic of 90 million people founded on a principle of democratic centralism; the government has been concerned with the regulation of alcohol since independence from Western colonial powers was declared in 1945. The legal provisions are dispersed in many pieces of legislation and are not well known or implemented throughout the country. Decree 94, passed in 2012, aims to regulate, over time, the production of informal alcohol and requires producers of home‐made alcohol to register with local authorities. The study was carried out in Hanoi, Thai Binh, Khanh Hoa and Dong Thap provinces (covering a population of four million).

In South Africa, a country of 55 million, and a constitutional democracy since 1994, alcohol has played a major role in colonial and apartheid history 8, 9. Prohibition for blacks, passed in 1897, was replaced by the development of revenue producing beer halls owned and run by the municipalities. These became sites of protests with women often at the forefront of the protest. It was also women who established shebeens, drinking places outside of the state system and places of independence as well as inebriation. The study was conducted in the City of Tshwane Metropolitan Municipality, located mainly within the province of Gauteng. It includes rural and metropolitan areas. The estimated population of Tshwane is 2 345 908 people.

### *Current alcohol consumption levels*

The countries differ in terms of gross domestic product, prevalence of alcohol use and estimated per capita levels of consumption (see Table 1). The countries also differ in the proportion of alcohol consumed that is estimated to be in the form of unrecorded (informal alcohol) with Vietnam in particular having a very high proportion of estimated consumption in the form of traditional ‘ruou trang’ (white spirits made from rice). In South Africa, consumption of informal alcohol, most in the form of traditional beer made from sorghum, has declined.

**Table 1:** Gross domestic product, prevalence of alcohol use and per capita levels of absolute alcohol consumption across countries

	Gross domestic product/capita 2013 (2011 PPP$)^a^	Prevalence of alcohol use: percentage consuming in past 12 months (2010 data)^b^	Total (and unrecorded) per capita (15+) consumption (litres of pure alcohol) (2008–2010)^b^	Per capita (recorded and unrecorded, 15+) consumption of drinkers only (litres of pure alcohol) (2008–2010)^b^
UK^c^	$37 017	83.9	11.6 (1.2)	13.79
New Zealand	$32 808	79.5	10.9 (1.6)	13.70
St. Kitts and Nevis	$20 709	42.5	8.2 (0.5)	19.31
Thailand	$13 932	29.7	7.1 (0.7)	23.83
South Africa	$12 106	40.6	11.0 (2.9)	27.09
Vietnam	$5 125	38.3	6.6 (4.6)	17.90

a United Nations Development Programme Human Development Report statistical annex.

b World Health Organization. Global Information System on Alcohol and Health. Available at: http://apps.who.int/gho/data/?showonly=GISAH&theme=main (accessed July 2016).

c Data are not available from this source for Scotland alone.

## Results

### *Availability*

#### *Restrictions on density and location*

Restrictions on outlet density were not universal (Table 2). They were reported in non‐metropolitan areas of Scotland, at the discretion of the licensing board, and in St. Kitts and Nevis and Vietnam.

**Table 2:** Restrictions on the density and location of alcohol outlets and outlet density per 1000 population

	Scotland	New Zealand	St. Kitts and Nevis	Thailand	South Africa	Vietnam
*Density of alcohol outlets restriction* a						
Metro on license	No	No	Yes	No	No	Yes
Metro off license	No	No	Yes	No	No	Yes
Non‐metro on license	No	No	Yes	No	No	Yes
Non‐metro off license	Yes	No	Yes	No	No	Yes
*Outlet restriction from certain locations* b						
	Yes	No	Yes	Yes	Yes	Yes
*Density of alcohol outlets per 1000 population*						
On‐premise						
Licensed	2.2	2.3	N/A	—	1.2	N/A
Licensed + unlicensed	—	—	N/A	9.0^c^	4.7	N/A
Off‐premise						
Licensed	0.9	1.0	N/A	—	0.4	1.0
Licensed + unlicensed	—	—	N/A	2.3^c^	0.6	3.5

a Is the density of on‐/off‐premise alcohol outlets restricted in any way (e.g. not situated within 1 km of another outlet).

b Are alcohol outlets restricted from certain locations, for example near temples, schools or public parks?

c In Thailand by law the licensing system does not categorise outlets into on‐/off‐premise. The estimate based on the observational survey includes unlicensed outlets.

N/A, not available; —, not applicable.

Locality restrictions were reported in all countries except New Zealand and included prohibition in relation to exposure to children (Scotland, Thailand and South Africa), places of worship (Thailand, South Africa and Vietnam), public parks (Thailand) and residential districts (South Africa), but in many cases it was reported these were not complied with.

#### *Density of outlets*

Estimates of density of outlets per 1000 population (Table 2) were taken from official licensing figures in the case of New Zealand, Scotland and South Africa (with the addition of estimates of unlicensed premises). Vietnam estimated its density, having no official data and Thailand reported based on an outlet survey. Density in Scotland and New Zealand was very similar at approximately 2 per 1000 on‐premise and 1 per 1000 off‐license. South Africa's outlets, largely unlicensed and on‐premise, were estimated at almost 5 per 1000 and Vietnam's, largely off‐premise and unlicensed, were 3.5 per 1000. Thailand does not distinguish off‐ and on‐premise by law; however, the survey showed the highest level, a total of 11.3 per 1000 population (9.0 for off‐premise and 2.3 for on‐premise).

Estimates of unlicensed outlets were made in New Zealand, which reported very few; Thailand (based on an outlet survey) reported 18% unlicensed. Vietnam estimated three to four times the allowed number of premises were trading. Similarly, South Africa estimated the majority of outlets were unlicensed. In St. Kitts and Nevis, the number of unlicensed off‐premise alcohol outlets was difficult to determine.

#### *Trading hours*

Many of the countries had some restrictions relating to particular days, including religious holidays and, in the case of Thailand, and St. Kitts and Nevis elections. The typical trading hours reflected the officially licensed hours in Scotland and New Zealand (although a survey of trading hours in New Zealand showed actual hours were influenced by demand from customers and could be shorter) 10. In Scotland, typical hours in both on‐and‐off licenses were approximately 12 h a day and in New Zealand somewhat longer at 16–18 h a day. South Africa's hours were approximately 11 h from off‐premise and 16 in on‐premise and could be longer on the weekends in shebeens. St. Kitts and Nevis and Vietnam hours were also affected by availability from informal sources and alcohol could be available 24 h a day. Thailand appeared to have the strongest restrictions in place with convenience stores trading for 10 h a day and on‐premise licenses for 7 h.

#### *Minimum purchase age*

All countries had established a minimum purchase age and in five of the six it was 18 years, with Thailand having a minimum purchase age of 20 years. In none of these countries did minimum purchase age differ by beverage or place of purchase.

#### *Social supply*

Legislative restrictions on the social supply of alcohol to those who are underage were in place in Scotland, South Africa, Thailand and New Zealand. No such restrictions were in place in St. Kitts and Nevis or Vietnam.

#### *Sale to intoxicated patrons*

Most countries (the exception was Vietnam) had restrictions around the sale of alcohol to intoxicated patrons.

#### *Marketing and sponsorship*

Thailand had relatively comprehensive legislation in place to restrict alcohol marketing including a ban on images of the product in advertising and a partial, time related, ban on showing corporate logos (Table 3); however, this could only control domestic sources of broadcast and digital marketing. In Thailand, fiscal promotions (discounted prices and free samples) were prohibited and alcohol industry sponsorship was banned in public stadiums and academic institutes. Vietnam also had legislation in place but this only covered alcohol beverages above 15%, meaning promotion of beer was permitted. It was also reported there was widespread promotion of all alcohol in digital marketing and in below the line activities (point‐of‐sale, associations with events and product placement). In Scotland, legislation banned promotion of price discounting (e.g. multi‐buy promotions) and there were partial restrictions on point‐of‐sale outlet promotions. New Zealand had similar restrictions on point‐of‐sale marketing on‐premise, but neither had regulations on mass media advertising and sponsorship of sporting events. In New Zealand, legislation banned promotions that were likely to encourage persons in on‐premise venues to consume alcohol to an excessive extent. There were no restrictions on advertising in the mass media. South Africa and St. Kitts and Nevis had the most permissive alcohol‐marketing situation with no legislation in place.

**Table 3:** Marketing and sponsorship legislative restrictions

	Scotland	New Zealand	St. Kitts and Nevis	Thailand	South Africa	Vietnam
*Country policies*						
Marketing/advertising restrictions	Ban price discounts, irresponsible marketing at point of sale	Ban on advertising promoting excessive consumption on‐premise	None	Total ban product images, Partial ban corporate ad 5 am to 10 pm, Ban sale promotions	None	Total ban spirits. Partial ban wine. Voluntary code beer
Sponsorship restrictions	None	None	None	Banned in public stadiums and academic institutes	None	Total ban spirits. Partial ban beer and wine

#### *Drink‐driving regulations*

All of the participating countries had drink‐driving legislation in place. In Thailand, South Africa and Vietnam, the legal maximum blood alcohol concentration (BAC) above which it was illegal to drive was 50 mg of alcohol per 100 mL (0.05/100) of blood (Table 4). For Scotland, New Zealand and St. Kitts and Nevis, the legal BAC was 0.08/100. New Zealand was the only country to have a differing BAC level for youth drivers, that is under 20 years no alcohol at all is allowed.

**Table 4:** Drink‐driving legal limits and mode of enforcement

	Scotland	New Zealand	St. Kitts and Nevis	Thailand	South Africa	Vietnam
*Country policy—legal maximum BAC* a *(g/100 mL)*						
Adult	0.08/100	0.08/100	0.08/100	0.05/100	0.05/100	0.05/100
Youth	0.08/100	0	0.08/100	0.05/100	0.05/100	0.05/100
*Enforcement methods*						
Random testing (stationary checkpoints)	No	Yes	No	Yes	Yes	Yes
Random testing (mobile patrol units)	No	Yes	No	Yes	Yes	Yes
Selective testing	Yes	Yes	Yes	Yes	Yes	Yes

a BAC, blood alcohol concentration.

All of the countries had at least one method for measuring and enforcing BAC levels (breath, blood or observational) (Table 4). South Africa and Thailand sometimes relied on observational measures (e.g. walking a straight line), but breath and blood testing were also used. Stationary roadside checkpoints, mobile patrol units and random testing were employed in New Zealand, Vietnam and South Africa. Thailand used sobriety check points and implemented random testing in metropolitan areas. Scotland and St. Kitts and Nevis do not have random breath testing but use selective testing where some grounds for suspicion are present.

#### *Key informants perception of enforcement of regulations and availability of alcohol*

The rankings of enforcement of regulations varied between countries with Scotland and New Zealand reporting higher levels of enforcement in many areas compared with the other participating countries (Table 5). Thailand reported a general picture of higher levels of enforcement compared with St. Kitts and Nevis, South Africa and Vietnam, with the exception that St. Kitts and Nevis reported high levels of enforcement of drink‐drive legislation. The restriction of social supply and sale to intoxicated patrons was less well enforced compared with the regulations on sale more generally.

**Table 5:** Key informants perceptions of enforcement and availability

	Scotland	New Zealand	St. Kitts and Nevis	Thailand	South Africa	Vietnam
*Perceptions of enforcement*						
Off‐premise sale of alcohol						
Enforcement^a^	8	9	5	5	5	2
On‐premise sale of alcohol						
Compliance^a^	9	8	2	6	5	3
Enforcement^b^	7	9	2	6	4	3
Minimum purchase age						
Compliance^a^	8	8	4	6	4	1
Enforcement^b^	8	9	3	6	4	1
Restriction of social supply						
Compliance^a^	4	4	N/A	5	2	N/A
Enforcement^b^	1	4	N/A	3	1	N/A
Restriction of sale to intoxicated patrons						
Compliance^a^	3	6	4	3.5	4	N/A
Enforcement^b^	3	7	3	3.5	3	N/A
Advertising and sponsorship restrictions						
Enforcement^b^	8	8	N/A	7	N/A	7
Sponsorship restrictions						
Enforcement^b^	N/A	N/A	N/A	5	N/A	N/A
Enforcement of drink‐driving restrictions						
Enforcement^b^	4	**—**	10	6	**3**	7
*Perceptions of availability*						
Off‐premise sale of alcohol						
Availability^c^	9	10	10	10	10	9
On‐premise sale of alcohol						
Availability^c^	7	10	10	10	10	9.5
Underage ease of purchase^d^	6	3	7	6	6	9.5
Ease of access by social supply^c^	7	8	8	7	9	8
Ease of purchase by intoxicated patrons^d^	7	6	7	8	9	7

a From a scale of 1 (completely ignored) to 10 (complete compliance).

b From a scale of 1 (not enforced) to 10 (always enforced).

c From a scale of 1 (completely unavailable) to 10 (completely available), how available are alcohol beverages?

d From a scale of 1 (not at all easy) to 10 (very easy).

Inadequate resource allocation led to insufficient policing which was highlighted as a major problem. Furthermore, drinking to intoxication was generally accepted and adherence to regulations not always highly regarded. In South Africa, police officers or inspectors have been known to accept bribes from liquor establishments, and a large proportion of the unregulated market (e.g. shebeens) did not comply with sale of alcohol policies or restrictions. In South Africa, there was no system for recording offenses against sale restrictions, and often the key informants themselves were unaware of regulations in place. There were few restrictions on home‐brew in South Africa as it was considered a part of tradition and culture. In Vietnam, while some establishments may refuse to sell alcohol products to intoxicated patrons, the key informants stated that the refusal was aimed at avoiding possible trouble by the customer.

Ease of purchasing alcohol while under the minimum purchase age was perceived as being relatively easy by all countries except New Zealand. Key informants in Vietnam stated there was no enforcement of the minimum purchase at all and that owners and staff of venues very rarely checked patrons’ ages and were sometimes ignorant of purchase age regulations, selling alcohol to almost anyone with the money to purchase it. Money was also cited as a factor in selling to underage patrons in South Africa, and parents sent children to liquor outlets to purchase alcohol on their behalf. Inadequate resource allocation to enforce minimum purchase ages was cited as a barrier in St. Kitts and Nevis.

The limited regulations on marketing in New Zealand and Scotland and somewhat broader restrictions in Vietnam and Thailand were reported to be fairly well enforced but in Vietnam it was noted alcohol manufacturers and importers sent advertising workers to discos, bars and restaurants where young people drink and the internet, particularly social media, showed adverts for spirits. In the only country reporting regulation of sponsorship (Thailand), enforcement was relatively low.

Regardless of the specifics of regulation and enforcement the key informants’ perception of general availability of alcohol was very high, with almost all of the ratings either 9 or 10, but assessments of ease of purchase and social supply to underage and ease of purchase by intoxicated were a little lower across the countries.

Enforcement of drink‐driving laws was perceived as being relatively low by the key informants in Scotland, South Africa and Vietnam. Bribery and corruption of traffic/police officials was cited by the key informants as the main reason for non‐enforcement in South Africa and in Vietnam a lack of equipment and personnel was reported. In St. Kitts and Nevis, a high level of enforcement was reported. Key informant data on drink driving were not collected in New Zealand, but enforcement is high 11, 12.

## Discussion

Results indicate the complexity of alcohol policy environments across countries and difficulty of assessing it by addressing only the legislative situation, not taking into account the enforcement and the extent of the informal market. Differences in legislative restrictions on availability through density and hours of trading are not reflected in key informants’ estimates of availability that were high in all countries. Where enforcement was perceived to be high, as in New Zealand, the policy with regard to availability was fairly liberal; conversely, where policy was more restrictive, as in the case of St. Kitts and Nevis and Vietnam (which had legislated restrictions on density) enforcement was perceived to be low and availability consequently high. High levels in Vietnam reflected availability of informal alcohol. Thai key informants also reported the highest level of availability and this was reflected in their observational survey results. It is possible density of outlets in South Africa would show higher levels than the current estimates if an observational survey was carried out.

Policy attempting to restrain youth access was present in all countries but differed in the extent to which it was implemented. Minimum purchase age was legislated in all countries, but more likely to be enforced in the higher income countries (excluding St. Kitts and Nevis). A study carried out in Thai off‐premise consumption stores showed very low levels of enforcement 13. Restrictions on social supply to young people were less commonly in place across countries and not well enforced leading to perceptions that young people would access alcohol more readily through social supply than purchasing it themselves.

Sale to intoxicated persons was also not well enforced, even in high‐income countries, and perceptions were of fairly easy access by intoxicated people. Low enforcement was often attributed to a lack of resources, a lack of awareness by the retailers that certain alcohol policies were in place, revenue as a factor driving sales to intoxicated person and acceptance of bribes by police and enforcement officers to overlook any non‐compliance of alcohol policy.

Alcohol marketing was largely unrestricted in most countries. The majority relied on voluntary codes administered by the producers and marketers of alcohol in which codes relating to exposure and/or content are publicised. These have been shown to be ineffective 4, 14. Where legislation did exist in high‐income countries this focused on point‐of‐sale advertising. The introduction in Scotland of restrictions on price promotions were relatively new, but had resulted in a 2.6% decrease in off‐trade alcohol sales compared to England and Wales by September 2012 15. Thailand was the exception in the study in having fairly comprehensive restrictions on marketing, including efforts to control social media marketing, but this was necessarily restricted to that originating from inside Thailand. Research has shown some failures of enforcement and comprehensiveness of the regulations 16. Vietnam had legislation prohibiting the promotion of higher potency beverages, although this was widely circumvented and promotion of beer, which is the commercial beverage rapidly expanding in use in the country 17, is unrestricted.

All countries had a per se law in place for drink‐driving and countries varied in level of enforcement with New Zealand having a clear focus on random breath testing. In other locations, either the less effective approach of selective testing was taken or there was a lack of enforcement resources being deployed. St Kitts and Nevis’ approach of selective testing was judged well enforced possibly reflecting the small size of the population.

The perceptions of key informants regarding policy enforcement provided a useful data source. However, this could be affected by the composition of the sample, for example non‐governmental organisations might have a different perception from those working in the enforcement sector and also by the number of key informants interviewed, which varied in this study reflecting different availability of resources. Overall, availability was judged at a similarly high level across all countries and it is unknown the extent to which key informants make judgements that can be compared between countries. However, as discussed above, there did seem to be a pattern in which strict legislation, in middle‐income countries, was not well enforced and where enforcement was more effective the policy itself was more liberal. Also, key informants from different countries did report differently in relation to other policy areas, for example enforcement of drink‐driving legislation, suggesting discrimination on their part.

Data availability varied between countries and reflected, in some cases, low level of development of the policy area. Observational and small‐scale research projects can be used to fill these data gaps. Some countries participating in the AEP included observational surveys and it would be useful in future use of the AEP to expand this data collection and ensure comparability as far as possible. In the current data collection, New Zealand found, following a phone survey of retail outlets, actual trading hours were fewer than licensed (as has been reported in a UK evaluation of an extension of trading hours 18) and Thailand found very high levels of unlicensed premises. The use of these approaches within the AEP enhances the value of the AEP in low and middle income countries.

These countries represent different stages in the replacement of informal alcohol by commercial, expansion of the drinking population and policy response. In only one middle‐income country, Thailand, was there an attempt to significantly restrain the alcohol marketing by the transnational alcohol corporations. The enforcement of legislation and regulations was also lower in the countries where this transition was taking place, that is Vietnam and South Africa. The high levels of unlicensed distribution channels with estimated very high levels of availability have implications for the spread and heavy consumption of commercial alcohol as it replaces informal alcohol.

Since the time of data collection in this study there have been some policy changes. Scotland and New Zealand have both reduced their BAC levels to 0.05 and New Zealand passed a *Sale and Supply of Alcohol Act* that increases restrictions on availability. Policy change is being given serious consideration in South Africa including a ban on most alcohol marketing except at points of sale and further controls on availability. Legislation is under development in Vietnam. The availability of the baseline data collected using the AEP will provide an opportunity to evaluate the effects of these policy changes.

## Conclusion

In countries with fewer resources, alcohol policies are less effective because of lack of implementation and enforcement and, in the case of marketing, lack of regulation. This has implications for the increase in consumption as a result of the expanding distribution and marketing of commercial alcohol and consequent increases in alcohol‐related harm. The AEP provides a tool to assess policy environments suitable for use in middle‐income countries.

## Conflict of interest

There are no competing interests to declare.
